# Reductions in sleep quality and circadian activity rhythmicity predict longitudinal changes in objective and subjective cognitive functioning in women treated for breast cancer

**DOI:** 10.1007/s00520-021-06743-3

**Published:** 2021-12-26

**Authors:** Sonia Ancoli-Israel, Lianqi Liu, Loki Natarajan, Michelle Rissling, Ariel B. Neikrug, Shawn D. Youngstedt, Paul J. Mills, Georgia R. Sadler, Joel E. Dimsdale, Barbara A. Parker, Barton W. Palmer

**Affiliations:** 1 Department of Psychiatry, University of California, San Diego, 9500 Gilman Drive, La Jolla, CA 92093-0737 USA; 2 University of California, San Diego Moores Cancer Center, San Diego, CA USA; 3 Department of Medicine, University of California, San Diego, San Diego, CA USA; 4 Family Medicine and Public Health, University of California, San Diego, San Diego, CA USA; 5 Department of Psychiatry and Human Behavior, University of California, Irvine, Irvine, CA USA; 6 Edson College of Nursing and Health Innovation, Arizona State University, Phoenix, AZ USA; 7 Department of Surgery, University of California, San Diego, San Diego, CA USA; 8 Veterans Affairs San Diego Healthcare System, San Diego, CA USA

**Keywords:** Cognitive function, Circadian activity rhythms, Sleep quality, Fatigue, Depression, Breast cancer, Chemotherapy

## Abstract

**Purpose:**

To examine long-term cognitive effects of chemotherapy and identify predictors among women with breast cancer (WBC).

**Patients and methods:**

Sixty-nine WBC scheduled to receive chemotherapy, and 64 matched-controls with no cancer, participated. Objective and subjective cognition, total sleep time, nap time, circadian activity rhythms (CAR), sleep quality, fatigue, and depression were measured pre-chemotherapy (Baseline), end of cycle 4 (Cycle-4), and one-year post-chemotherapy (1-Year).

**Results:**

WBC showed no change in objective cognitive measures from Baseline to Cycle-4 but significantly improved from both time points to 1-Year. Matched-controls showed an increase in test performance at all time points. WBC had significantly higher self-reported cognitive dysfunction at Cycle-4 and 1-Year compared to baseline and compared to matched-controls. Worse neuropsychological functioning was predicted by less robust CARs (i.e., inconsistent 24 h pattern), worse sleep quality, longer naps, and worse cognitive complaints. Worse subjective cognition was predicted by lower sleep quality and higher fatigue and depressed mood.

**Conclusion:**

Objective testing showed increases in performance scores from pre- and post-chemotherapy to one year later in WBC, but matched-controls showed an increase in test performance from baseline to Cycle-4 and from Cycle-4 to 1-Year, likely due to a practice effect. The fact that WBC showed no practice effects may reflect a form of learning deficit. Compared with the matched-controls, WBC reported significant worsened cognitive function. In WBC, worse objective and subjective cognitive functioning were predicted by worse sleep and sleep-related behaviors (naps and CAR). Interventions that target sleep, circadian rhythms, and fatigue may benefit cognitive function in WBC.

## Introduction

Chemotherapy can produce reports of acute cognitive dysfunction [1–3] with one-third of patients with cancer reporting long-term impairment lasting up to five years or more [1, 3]. In addition, those receiving high-dose chemotherapy or concurrent cytotoxic agents have more cognitive disruption than those on standard doses [4, 5]. The deleterious cognitive effects, both objective and subjective, persist even after adjusting for variance attributable to depression, age, education, and premorbid IQ [6, 7]. While there is no specific cognitive profile characterizing the effects of chemotherapy, chemotherapy appears to affect multiple cognitive domains including attention/concentration and working memory, episodic learning and memory, mental processing/psychomotor speed, and executive function [8–10]. Although the cognitive decline may be subtle, it is common and can have a deleterious impact on quality of life (QOL) [1, 8, 11]. Imaging studies confirm that chemotherapy induces functional and/or structural changes in the brain which are associated with worse cognitive performance [12–14], providing fundamental evidence supporting these findings. Nevertheless, cognitive dysfunction is rarely diagnosed or addressed in patients with cancer.

A major question is whether there are modifiable predictors of cognitive dysfunction in patients with cancer. Being able to identify predictors could lead to preventive measures. Impairments in CARs have been linked with prospective cognitive decline in older adults [15], and worsening of CAR synchronization during chemotherapy for breast cancer is associated with reported declines in cognitive function [16]. Moreover, impairments in cognitive function have been linked with poor sleep in breast cancer [17, 18]. Our previous research with these same WBC revealed a wide range of circadian synchronization and a wide range of sleep.[19]. Therefore, the current study explored whether decreases in robustness of CAR and sleep quality and increases in fatigue predict decreases in cognitive functioning, as these factors have been predictive of other outcomes in cancer, including survival [20], QOL [20, 21], and markers of cancer progression [22].

Consensus guidelines suggest that studies include baseline or pre-chemotherapy treatment assessments, longitudinal or long-term follow-up, and standardized use of measures [23]. Inclusion of subjective assessment of cognitive decline is also encouraged as it is associated with patient QOL [24] and possibly with treatment adherence. The present study addressed these issues by collecting data before, during and one-year after chemotherapy and by using objective and subjective assessments of cognition. Such longitudinal studies with pre-, post-chemotherapy, and subsequent follow-up measurements may elucidate whether the cognitive changes are preventable or reversible following the end of chemotherapy.

Our primary hypothesis was that cognitive performance will decrease from pre-chemotherapy to the fourth cycle of chemotherapy in WBC relative to matched-controls but will remain stable from cycle four to one-year follow-up. Our second hypothesis was that WBC experiencing worsening CAR, poor sleep, and fatigue (after controlling for depression) would exhibit decreases in cognitive function compared to baseline.

## Methods

### Participants

**Fig. 1:**
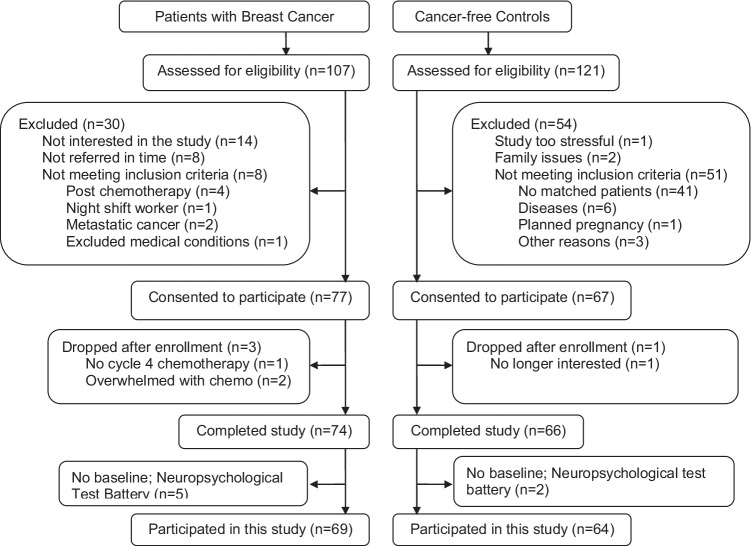
Screening and enrollment flowchart

Women (*n* = 107) with newly diagnosed stage I-III BC scheduled to receive at least four cycles of adjuvant or neoadjuvant chemotherapy were referred by oncologists in the San Diego area, primarily from the UC San Diego Moores Cancer Center. Each was screened for inclusion and 69 were enrolled in the study (see Fig. 1).

Each participant was asked to nominate a friend with no cancer for inclusion in the matched-control group. For those women with no adequately comparable friend or who did not wish to nominate a friend, a separate recruitment was performed to find demographically matched volunteers. Altogether, 121 women without a history of cancer were evaluated for inclusion in the control group based on closely matching to a specific cancer patient by age (within five years), ethnicity, and education. Sixty-four women with no cancer were included in this matched-control comparison group. Some data from participants in the present study were included in prior published reports [19, 25, 26]; however, the present report represents our first examination of the association of these measures with longitudinal cognitive tests.

Exclusion criteria included pregnancy, current bone marrow transplant, radiotherapy, metastatic breast cancer, confounding underlying medical illnesses, significant pre-existing anemia (Hg < 10 gm/dl), or other physical or psychological impairments. Exclusion criteria for the control group also included any history of cancer (other than non-melanoma skin cancers).

The study was approved by the UCSD Office of IRB Administration and by the UC San Diego Moores Cancer Center’s Protocol Review and Monitoring Committee. All women provided written informed consent before participation.

### Procedures

After HIPAA-waiver authorization was provided, medical records were abstracted for medical history and current medication use. Data were collected at three time points: before the start of chemotherapy (Baseline), at the end of cycle 4 chemotherapy (Cycle-4), and 1 year after the start of chemotherapy (1-Year). At each time point, a neuropsychological test battery, along with other questionnaires, was administered. Starting on the first day of each time point, women also wore an actigraph for three consecutive days (72 h) and completed a daily sleep log used for editing actigraphy data.

### Measures

#### Cognitive functioning

Objective cognitive functioning was examined with a neuropsychological test battery which included tests focusing on four domains of cognitive abilities suggested in prior literature as being the most sensitive to chemotherapy-related changes: (1) Episodic Learning/Memory, (2) Executive Function, (3) Verbal and Visual Attention/Working Memory, (4) Psychomotor/Mental Processing Speed [23]. The specific tests within each domain are shown in Table 1. In selecting the above battery, we endeavored to balance comprehensive assessment with participant burden [27]. In addition, where possible, we selected tests with multiple alternative forms (particularly HVLT-R and BVSM-R) to avoid practice effects from learned content over repeated testing. All tests were administered in the morning either at the participant’s home or at the clinic.

**Table 1:** Domains and tests used for the neuropsychological test battery

Domain	Test	Score used for analysis
Episodic Learning/Memory	Hopkins Verbal Learning Test – Revised (HVLT-R); Published parallel forms for the HVLT-R were used in fixed-order to practice effects from item content familiarity.	Total recall on learning trials 1 through 3
Executive Function	1. Trail Making Part B, 2. Wisconsin Card Sorting Test (WCST-64) 3. Color-Word Interference trial of the Stroop Color and Word Test 4. Letter Fluency (FAS) Test and Category (Animals) Fluency Test	1.Seconds to complete 2. Conceptual level responses 3. Total words completed 4. Total correct words generated
Verbal and Visual Attention/Working Memory	1. Digit Span of the Wechsler Adult Intelligence Scale - Third Edition (WAIS-III) 2. Digit Cancellation task	1. Raw score 2. Total correct
Psychomotor/Mental Processing Speed	3. Trail Making Test Part A 4. WAIS-III Digit Symbol and Symbol Search subtests 5. Stroop Color and Word Test	3. Time to complete (seconds) 4. Raw scores 5. Total words completed on the Color or Word trial

A summary measure of cognitive ability, a neuropsychological composite score, was computed as follows: each component raw test score was converted to a *z*-score by subtracting the baseline mean and dividing by the standard deviation. *Z*-scores were coded so that higher scores represented better functioning. The composite score was defined as the mean of *z*-scores over the entire battery.

Subjective cognitive functioning was assessed with the Patient’s Assessment of Own Functioning Inventory (PAOF) [28], a validated self-report questionnaire designed to elicit an individual’s ratings of her ability to function in everyday tasks. The 33-items cover areas of memory (Memory), language and communication (Language), sensory-perceptual and motor skills (Sensory-Motor), and higher level cognitive and intellectual function (Cognitive). The questions are rated on a 6-point scale from 0 (almost never) to 5 (almost always). A total score and scores for the four areas are calculated using Rourke’s method, with a higher score indicating more self-reported cognitive concerns [28]. Only total score results are presented.

#### Sleep

The Pittsburgh Sleep Quality Index (PSQI) [29] was used for subjective sleep quality. It measures reported sleep patterns and problems, including sleep quality, sleep latency, sleep efficiency, and napping behavior. The PSQI is a 19-item questionnaire that has been demonstrated to have high internal consistency (0.83), test-retest reliability (0.85), and diagnostic validity. The global score (range 0–21) reflects the overall quality of sleep over the prior one-week with high scores reflecting poor sleep quality.

Objective measures of sleep were obtained with wrist actigraphy. All women wore an actigraph for three consecutive days (72 h). The Actillume-II (Ambulatory Monitoring Inc, Ardsley, New York) and the Actiwatch-Light (Philips Respironics Mini Mitter, Bend, OR) were used. The Actillume-II is a small device approximately 1 × 3 × 6 cm in size, containing a piezoelectric linear accelerometer (sensitive to 0.003 g and above), a microprocessor, 32K RAM memory, and associated circuitry. The Actiwatch-Light is a watch-like device approximately 1 × 2.5 × 5 cm in size, also containing a piezoelectric linear accelerometer (sensitivity < .01 g-force) with a sampling rate of 32 Hz to measure and record wrist movement. Once collected, data were downloaded onto a desktop computer and information from a sleep log which recorded time to bed, time up in the morning, nap time was used for editing. The Action-4 software for Actillume-II and Actiware-5 software for Actiwatch-Light were used to score sleep and wake. A one-minute epoch setting was used for both devices. For the sleep/wake analysis, the SUMACT (summary activity) channel was used for Actillume, and the default (medium) activity sensitivity threshold was set for the Actiwatch-Light. The following parameters are reported in this study: total sleep time (TST), wake after sleep onset (WASO), percent sleep (%sleep), and total nap time (NAPTIME). Percent sleep was defined as the amount of sleep between sleep onset and final awakening. This is similar to sleep efficiency except that the starting time point is sleep onset and not lights out (since lights out is less reliable with actigraphy) and so sleep onset latency is not included in the equation. A nap was defined as at least ten continuous minutes of inactivity (i.e., sleep) during the out-of-bed period.

As previously published [19, 25, 26], 13 WBC and 7 women with no history of cancer wore an Actillume-II, and the remainder wore an Actiwatch-Light. A validation study showed high correlation between the two (all *r’s* > 0.85), and therefore the data from the two devices were deemed equivalent.

For each participant, actigraphy began on the same day at each time point. The day chosen was based on the day of chemotherapy administration. While the ideal recording time for an actigraph is generally one week, due to potential subject burden, the minimum of three days suggested by the AASM practice parameters for actigraphy [30] was used in this study.

#### Circadian activity rhythms

**Fig. 2:**
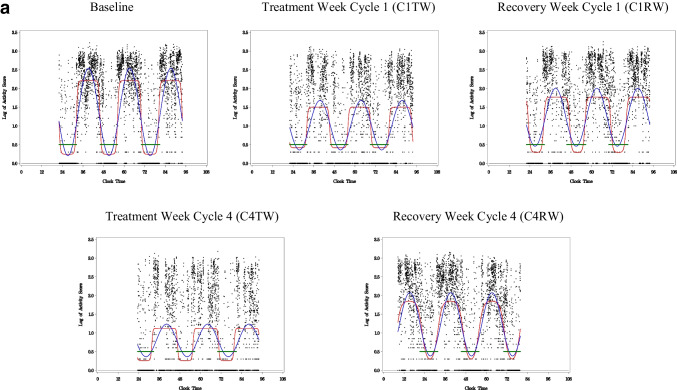
Example of a weak CAR. This woman with BC showed a robust sleep-wake rhythm at baseline indicated by clear contrast between daytime and nighttime activity. The rhythm then became disrupted during the first week of chemotherapy treatment (C1TW) and remained disrupted for the duration of chemotherapy, as indicated by lower amplitude and less contrast between bedtime and wake time. *X*-axis is clock time. Y-axis is log value. Black dots, log of activity scores calculated by the actigraphy. Blue line, best-fitting traditional cosine curve. Red line, extended cosine curve. Green line, in-bed time

**Fig. 3:**
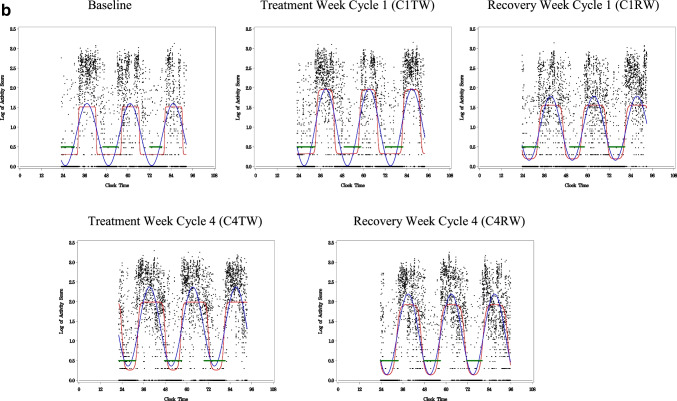
Example of a robust CAR. This woman with BC showed a robust sleep-wake rhythm at baseline, indicated by clear contrast between daytime and nighttime activity. This patient’s rhythm remained overall stable and robust throughout chemotherapy. *X*-axis is clock time. *Y*-axis is log value. Black dots, log of activity scores calculated by the actigraphy. Blue line, best-fitting traditional cosine curve. Red line, extended cosine curve. Green line, in-bed time

The activity level per epoch (minute) measured by actigraphy was used to estimate CAR. A robust CAR would be indicated by a clear contrast between daytime and nighttime activity. This patient’s rhythm would remain stable and robust throughout chemotherapy. A disrupted rhythm would be one with lower amplitude and less contrast between daytime and nighttime activity as compared to a rhythm of good rhythmicity, in this case, the comparison to baseline (see Fig. 2). CARs were analyzed by fitting each subject’s activity data to a 5-parameter extended cosine model, an antilogistic transformation of the standard cosine curve, allowing for estimation of parameters describing the shape of the 24-h rest/activity rhythm [31]. A series of variables could be calculated from this model, including circadian activity rhythmicity (R-squared} [32]. R-squared is the reduction in squared error which results from using a model to summarize data (and predict future values) compared to the mean [32]. Data sets that are most rhythmic or have the strongest rhythm yield the largest R-squared. The R-squared is reported in the study as the measurement of circadian activity rhythmicity. Figures 2 and 3 show examples of weak and robust circadian activity rhythmicity.

#### Fatigue

The 30-item Multidimensional Fatigue Symptom Inventory-Short Form (MFSI-SF) was used to measure fatigue [33], which resulted in five subscales/factors: General, Physical, Emotional, Mental, and Vigor. Each subscale includes six items, and each item is rated on a 5-point scale indicating how true the statement was during the last week (0 = not at all, 4 = extremely). The sum of General, Physical, Emotional, and Mental subscale scores minus the Vigor subscale score generates a total score. Range of possible scores for each subscale is 0 to 24, and the range for total score is − 24 to 96, with higher score indicating more severe fatigue, except for the Vigor subscale, where a larger score indicates less fatigue.

#### Depressive symptoms

Depressive symptoms were measured with the Center of Epidemiological Studies-Depression (CES-D) [34], a 20-item Likert scale of depressive symptoms based on the degree to which symptoms were experienced during the last week. This scale has been shown to have high reliability and validity in the assessment of depressive symptoms [34]. Since the CES-D reflects cognitive and affective symptoms rather than somatic symptoms of depression, it is highly recommended for use with patients with medical problems.

### Data analysis

Descriptive statistics (mean, standard deviation, and standard error) were calculated for all outcomes at all three time points, and group differences (WBC vs matched-controls) were evaluated with *t*-tests and chi-square tests as appropriate.

A mixed model analysis was used to examine the changes in neuropsychological tests and total PAOF score from Baseline to Cycle-4 and 1-Year, with group and time included as fixed covariate effects. Baseline was the reference time point and the matched-control group was the reference group. A group-by-time interaction was tested; significance of the group-by-time term would indicate the changes from Baseline between the two groups were significantly different. A random intercept was included in each mixed model to account for subject-specific effects. Further post hoc tests were conducted using appropriate contrasts: between group differences at each time point, and/or within group changes between any two time points.

Finally, in order to explore predictors of changes in objective and subjective cognitive functioning in the WBC, a series of mixed models were fit with the neuropsychological composite or domain score, or the total PAOF score, as the repeated measures dependent variable, i.e., a vector comprising the Baseline, Cycle-4 chemotherapy, and 1-Year values of the outcome. The independent variables, assessed in separate models, included subjective cognitive functioning (total PAOF score) or objective cognitive functioning (composite score), sleep (PSQI, TST, WASO, %sleep and NAPTIME), fatigue, depressive symptoms, and the circadian activity rhythmicity (R-squared). Leveraging the availability of longitudinal predictors (i.e., sleep, fatigue etc), we included the independent variables as time-varying covariates in the mixed models with the tested predictor included as a random effect in each mixed model, thereby allowing for subject-specific slope terms for this particular predictor in the model. Time was modeled as a fixed (categorical) effect representing Baseline (reference), Cycle-4, and 1-Year. Since objective and subjective cognitive functioning were correlated with age and education, the age and education (with or without a college degree) were adjusted for these two variables as co-variates in all mixed models; additionally, since BMI was also correlated with total PAOF score, BMI was adjusted in all PAOF models. Thus, our marginal model can be written as Y_ij_ = β_0_ + β X_ij_ + β_2_*covariates + error, where Y_ij_ and X_ij_ are the cognition outcome and predictor respectively for subject i at time j with the baseline time-point corresponds to j = 1, i.e., the reference; “covariates” refers to the covariates such as time, age, and education that were included in the model. Due to the inclusion of time-varying predictors, the coefficient β represents the (average) change in outcome (Y) per 1-unit increase in predictor X, and thus can be interpreted in our study as the average change (from baseline) in cognition score corresponding to a 1-unit increase (from baseline) in the predictor (e.g., total sleep time). The adjusted regression coefficients (ß-value) with standard errors and associated *p*-values of that model are presented.

All analyses were performed using version 9.3 of SAS (SAS Institute Inc. 2010).

## Results

### Demographics and participant characteristics

Demographics and disease characteristics are listed in Table 2. There were no significant differences between the two groups in age, BMI, race, education, marital status, household annual income, or baseline menopause status (all *p*’s > 0.16). There were also no significant differences in baseline levels of objective or subjective cognitive ability.

**Table 2:** Demographic and disease characteristics of women with breast cancer and women with no cancer (matched-controls)

Characteristics	Women with breast cancer (*n* = 69)	Matched-controls (*n* = 64)
Age (years)		
Mean (SD)	52.2 (9.5)	51.9 (9.3)
Range	31 – 80	29 – 81
Body mass index (BMI, kg/m^2^)		
Mean (SD)	27.5 (7.4)	26.9 (8.5)
Range	19.3 – 61.9	19.1 – 64.5
Race [n (%)]		
Caucasian	61 (88.4)	56 (87.5)
Non-Caucasian	8 (11.6)	8 (12.5)
Education [n (%)]		
Below competed college	35 (50.7)	23 (35.9)
Completed college and above	34 (49.3)	41 (64.1)
Marital status [n (%)]		
Never married	3 (4.3)	7 (10.9)
Divorced/separated/widowed	18 (26.1)	13 (20.3)
Married	48 (69.6)	44 (68.8)
Household annual income [n (%)]		
< $100,000	43 (62.3)	41 (64.1)
> $100,000	26 (37.7)	23 (35.9)
Baseline menopausal status [n (%)]		
pre-menopause	27 (40.9)	23 (37.7)
peri-menopause	7 (10.6)	8 (13.1)
post-menopause	27 (40.9)	22 (36.1)
hysterectomy	5 (7.6)	8 (13.1)
Not available	3	3
Cancer stage [n (%)]		-
Stage I	16 (23.5)	
Stage II	29 (42.7)	
Stage III	23 (33.8)	
Not available	1	
Surgery type		-
Lumpectomy	33 (47.8)	
Mastectomy	30 (43.5)	
Double mastectomy	3 (4.3)	
No surgery before Chemotherapy	3 (4.3)	
Chemotherapy regimen [n (%)]		-
AC	12 (18.2)	
AC + docetaxel	6 (9.1)	
AC + paclitaxel	34 (51.5)	
Other	14 (21.2)	
Not available	3	

There were no significant differences between the two groups in age, BMI, race, education, marital status, household annual income, and baseline menopause status (all *p*’s > 0.09). *AC* Doxorubicin + Cyclophosphamide

The trajectories of symptoms of sleep (subjective and objective), fatigue, depressive symptoms, and CAR over time were previously reported [19]. To briefly summarize, WBC spent more time napping and had more disrupted CARs and reported worse sleep quality, more fatigue, more depressive symptoms, and worse QOL than the matched-controls at baseline, cycle-4, and 1-Year. WBC also showed significantly worse sleep, increased fatigue, more depressive symptoms, and more disrupted CARs compared to their own baseline levels and to matched-controls at Cycle-4. At 1-year, these symptoms in the WBC returned to baseline levels but were still significantly worse than the matched-controls [19].

### Changes in objective measures of cognitive function over time

#### Composite score

**Fig. 4:**
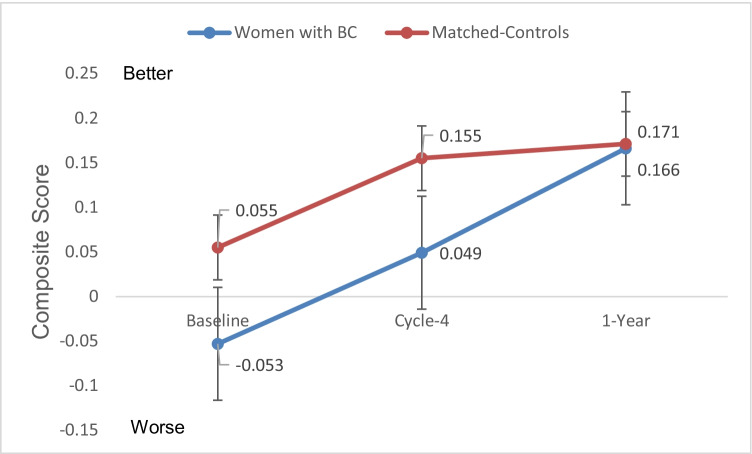
Neuropsychological test battery composite (mean+SE). Higher scores indicate better cognitive functioning. There was a significant time effect for WBC (*p* < 0.015) and for the matched controls (*p* < 0.001). Post hoc analysis showing that the neuropsychological composite score for WBC showed no change from Baseline to Cycle-4 (*p* = 0.330), but increased scores (i.e., improved scores) from Baseline to 1-Year (*p* = 0.004) and from Cycle-4 to 1-Year (*p* = 0.041). Matched controls (NC) scores increased (i.e., improved) from Baseline to Cycle-4 (*p* = 0.012) and 1-Year (*p* < 0.0001). There was no significant group effect (*p* = 0.22) or group-by-time interaction (*p* = 0.13). Age and education were controlled in the mixed models. NP Neuropsychological test battery. Baseline, before the start of chemotherapy; Cycle-4, at the end of cycle 4 chemotherapy; 1-Year, 1 year after the start of chemotherapy

The neuropsychological composite test results are shown in Fig. 4, with higher scores indicating better objective cognitive function. After adjusting for age and education, mixed model analysis for the composite score showed no significant group effect (*p* = 0.22) with post hoc analysis showing no significant differences between the two groups at baseline or at any of the three time points (all *p* > 0.14). There was a significant time effect for WBC (*p* = 0.015) with post hoc analysis showing that the neuropsychological composite score showed no change from Baseline to Cycle-4 (*p* = 0.330), but increased scores (i.e., improved scores) from Baseline to 1-Year (*p* = 0.004) and from Cycle-4 to 1-Year (*p* = 0.041). There was also a significant time effect for matched-controls (*p* < 0.0001) with post hoc analysis showing that the neuropsychological composite scores increased (i.e., improved) from Baseline to Cycle-4 (*p* = 0.001), from Baseline to 1-Year (*p* < 0.0001), and from Cycle-4 to 1-Year (*p* = 0.039). Similar results were found for Episodic Learning/Memory and Executive Function (data not shown).

### Changes in subjective measures of cognitive function over time

**Fig. 5:**
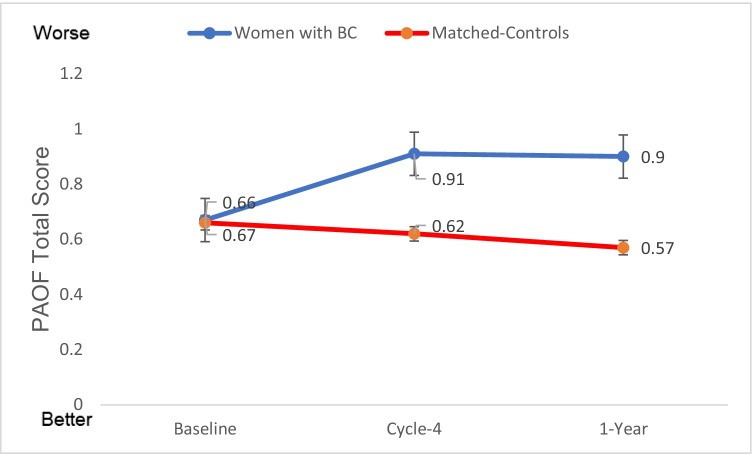
PAOF total score (mean+SE). Higher scores indicate more neurocognitive complaints. Analysis showed no difference between the groups at baseline (*p* = 0.79), but compared to matched-controls, WBC reported significantly higher total PAOF scores at Cycle-4 (*p* = 0.0054) and 1-Year (*p* = 0.022). There was also a significant time effect for WBC (*p* < 0.0001) with post hoc analysis showing that compared to Baseline, these women reported significantly higher scores at Cycle-4 (*p* < 0.001) and at 1-Year (*p* = 0.0008) than matched-controls. There was no significant time effect for the matched-controls (*p* = 0.59). However, there was a significant group-by-time interaction (*p* = 0.0005), with the increases from Baseline to Cycle-4 and 1-Year being significantly higher in WBC compared to the matched-controls (*p* < .001, *p* < 0.001, respectively. Baseline, before the start of chemotherapy; Cycle-4, at the end of cycle 4 chemotherapy; 1-Year, 1 year after the start chemotherapy. Total PAOF score: marginal group effect (*p* = 0.052), significant time effect for WBC (*p* < 0.0001), significant group-by-time interaction (*p* = 0.0005). Age and education were controlled in the mixed models

PAOF total scores are shown in Fig. 5, with higher scores indicating self-perception of worse functioning. After adjusting for variance attributable to age and education, mixed model analysis for the PAOF total score showed that the group effect was not significant (*p* = 0.052); post hoc analysis showed no difference between the groups at baseline (*p* = 0.79), but compared to matched-controls, WBC reported significantly higher total PAOF score at Cycle-4 (*p* = 0.0054) and 1-Year (*p* = 0.022). There was also a significant time effect for WBC (*p* < 0.0001) with post hoc analysis showing that compared to Baseline, these women reported significantly higher scores at Cycle-4 (*p* < 0.001) and at 1-Year (*p* = 0.0008) than matched-controls. There was no significant time effect for the matched-controls (*p* = 0.59). However, there was a significant group-by-time interaction (*p* = 0.0005), with the increases from Baseline to Cycle-4 and 1-Year being significantly higher in WBC compared to the matched-controls (*p* < .001, *p* < 0.001, respectively). These data suggest that, on average, WBC report decrements in cognitive function immediately post-completion of four cycles of chemotherapy and 1-Year later (compared to pre-chemotherapy) while women with no cancer report no marked changes. Similar results were found for the Language and Cognitive areas (data not shown).

### Predictors of objective and subjective cognitive functioning

As seen in Table 3, after adjusting for variance attributable to age, education, and time, mixed model results showed that the changes in the objective neuropsychological composite score were negatively associated with changes in total PAOF score, total PSQI score, and NAPTIME, and positively associated with changes in circadian activity rhythmicity (R-squared; all *p*’s < 0.05), indicating that a decrease in cognitive functioning (at follow-up compared to baseline) was predicted by changes resulting in less robust CARs, worsening self-assessment of cognitive ability, worsening sleep quality, and increases in nap time compared to baseline.

**Table 3:** Mixed model results with neuropsychological composite or domain score as the dependent variable and parameter of subjective assessment of cognitive ability (total PAOF), sleep, fatigue, depressive symptoms, or rhythmicity of circadian activity as the independent variable in separate models in women with breast cancer (*n* = 69)

Dependent variable	Independent variable	Mixed model results ^a^		
		Adj. ß-value	Standard error	*p*-value
Composite score	PAOF total score	-0.147	0.0513	**0.0052**
	Total PSQI score	-0.0212	0.00746	**0.0055**
	TST	0.0529	0.0272	0.055
	NAPTIME	-0.0586	0.0285	**0.042**
	WASO	0.01644	0.05062	0.75
	%sleep	0.1057	0.5202	0.84
	Total MFSI-SF score	-0.00259	0. 00150	0.088
	Total CES-D score	-0.00441	0.00275	0.11
	Circadian activity rhythmicity	0.467	0.207	**0.027**

^a^Adjusted for time, age, and college degree. *PAOF*, Patient’s Assessment of Own Functioning, higher scores indicate worse functioning; *PSQI*, Pittsburgh Sleep Quality Index, higher scores indicate poorer sleep quality. *TST*, nighttime total sleep time; *NAPTIME*, daytime total nap time; *WASO*, wake after sleep onset; *%sleep*, percent sleep from sleep onset to final wakening; *MFSI-SF*, Multidimensional Fatigue Symptom Inventory-Short Form, higher scores indicate more fatigue; *CES-D*, the Center of Epidemiological Studies-Depression, higher scores indicate worse depressive symptoms. Circadian activity rhythmicity is represented by R-squared, with a smaller number indicating a less robust circadian activity rhythm

As seen in Table 4, after adjusting for variance attributable to age, education, BMI, and time, mixed model results showed that the changes in subjective assessment of cognitive function (total PAOF score) were negatively associated with changes in neuropsychological composite score, and positively associated with changes in total PSQI score, total MFSI-SF score, and total CES-D score, indicating that compared to baseline, worsening objective cognitive functioning, sleep, fatigue, and depressive symptoms were associated with worsening self-reported cognitive function.

**Table 4:** Mixed model results with Subjective Cognitive Assessment (POAF) total score as the dependent variable and parameter of objective assessment of cognitive ability (composite score), sleep, fatigue, depressive symptoms, or rhythmicity of circadian activity as the independent variable in separate models in women with breast cancer (*n* = 69)

Dependent variable	Independent variable	Mixed model results ^a^		
		Adj. ß-value	Standard error	*p*-value
Subjective Cognitive Assessment (PAOF) total score	Composite score	-0.2378	0.09802	**0.0172**
	Total PSQI score	0.03601	0.01066	**0.0011**
	TST	-0.05832	0.05128	0.2583
	NAPTIME	0.04437	0.04323	0.3074
	WASO	0.02456	0.06949	0.72
	%sleep	-0.6974	0.6770	0.31
	Total MFSI-SF score	0.01553	0. 002256	**<0.0001**
	Total CES-D score	0.02539	0.003777	**<0.0001**
	Circadian activity rhythmicity	-0.3162	0.2971	0.2899

^a^Adjusted for time, age, and college degree. *PAOF*, Patient’s Assessment of Own Functioning, higher scores indicate worse functioning; *PSQI*, Pittsburgh Sleep Quality Index, higher scores indicate poorer sleep quality. *TST*, nighttime total sleep time; *NAPTIME*, daytime total nap time; *WASO*, wake after sleep onset; *%sleep*, percent sleep from sleep onset to final wakening; *MFSI-SF*, Multidimensional Fatigue Symptom Inventory-Short Form, higher scores indicate more fatigue; *CES-D*, the Center of Epidemiological Studies-Depression, higher scores indicate worse depressive symptoms. Circadian activity rhythmicity is represented by R-squared, with a smaller number indicating a less robust circadian activity rhythm

WASO and %sleep were not significantly associated with changes in either objective or subjective cognitive function.

## Discussion

This report describes a novel prospective study which included comprehensive objective and subjective cognitive assessment and a pre- and post-chemotherapy design with a 1-Year follow-up [35], to examine the short and long-term effects of chemotherapy on objective and subjective cognitive functioning among women treated for breast cancer [35] compared to a matched-control group. Predictors of cognitive change [2, 36, 37], specifically well-established and well-defined measures of objective and subjective sleep and circadian rhythmicity, were also examined. The results showed that declines in robustness of the CAR, sleep (specifically subjective reports of sleep quality and recorded time spent napping) from baseline to the end of cycle 4 and to 1-year later were consistently associated with declines in objective measures of cognitive function. Declines in objective measures of cognitive function, subjective sleep quality, fatigue, and depression from baseline to the end of cycle 4 and to 1-year later were consistently associated with declines in subjective reports of cognitive function. Changes in TST, WASO, and %sleep were not significantly associated with changes in either objective or subjective measures of cognitive function.

In WBC, objective neuropsychological testing showed no change in cognition at the end of cycle-4 chemotherapy compared to baseline, but there was an increase in performance scores from pre- and post-chemotherapy to one year later which is consistent with other findings [38]. On the other hand, matched-controls showed an increase in performance scores from baseline to the time point equivalent to the end of cycle-4, and continued to show increasing test performance at one year. The increases in performance scores seen in the matched-controls likely reflect practice effects, as is common with repeated testing due to increased task familiarity (even with use of alternate forms to avoid learning of specific content) [39]. The absence of improved test-performance with repeated testing among WBC from pre-chemotherapy to post-chemotherapy therefore requires explanation. Only one year later did they manifest some improvement in test performance. A failure to benefit from practice effects seen in these women may represent a form of learning deficit [40]. On the other hand, it is reasonable to consider that acute effects of chemotherapy might have contributed to poorer performance at Cycle-4, especially since the WBC increased their scores to those of the matched-controls at 1-Year. This type of pattern may suggest transient effects rather than the insidious decline more often observed in Alzheimer's Disease [41].

In a study examining white matter integrity in patients with cancer and survivors, time since treatment was negatively associated with less white matter integrity in the survivor group, suggesting microstructural integrity deterioration over time [14]. That idea, along with our finding that the WBC showed no practice effect on their objective neuropsychological testing, adds additional support to the idea that the impact on cognition may be in the ability to gain new knowledge and learning. Our results also support the value of having a non-patient group comparison for repeated neuropsychological tests [41].

While there was no difference in reports of subjective cognitive function between the two groups at Baseline, the WBC reported significantly more subjective cognitive impairment at both Cycle-4 and 1-Year. They also reported significantly more impairment compared to their own baseline levels. A review by Hutchison et al. on differences between patient-reported and objectively measured cognitive disabilities found that only 8 of 24 studies identified a significant relationship between the two, and suggested that this phenomenon might be explained by the differences in measurement methods and in definitions of impairment [42]. Likewise, Bray et al. attributed a low rate of correlation between objective and subjective cognitive measures to an inadequate assessment of cognitive function in many of the questionnaires [43]. Ganz et al. found that correlations between objective and subject measures of cognitive function were domain specific [10]

Consistent with our second hypothesis, the strongest predictors of objective cognitive change were changes in robustness of the CAR, sleep, and fatigue, after covarying for age, education, and time. Similar findings were also reported in other studies, with changes in cognition following chemotherapy for breast cancer being associated with baseline fatigue, depression and functional well-being [44], Depression and fatigue were found to account for brain activation differences which resulted in changes in cognitive function [6]. However, our study is unique in that we evaluated *longitudinal* effects, i.e., how changes in predictors impacted changes in cognition.

The present finding that the neuropsychological function was predicted by the robustness of the CAR is consistent with evidence that less robustness is predictive of cognitive decline in older adults [45] and associated with Alzheimer’s Disease [46]. However, to our knowledge, this is the first study to report these results in patients with cancer.

Since this was an observational study, the results do not definitely establish that impairments in sleep and circadian synchronization cause declines in cognitive function. However, multiple studies have shown that experimentally induced sleep impairment [47] and circadian misalignment elicit impairments in cognitive function [48]. Conversely, interventions to promote sleep (e.g., cognitive behavioral treatment for insomnia; CBT-I) and circadian synchronization (e.g., bright light) have elicited improvements in cognitive function and fatigue or kept them from getting worse in breast cancer [49, 50]. Further research exploring the benefits of CBT-I and bright light is warranted for patients with breast cancer and other types of cancer.

The current study had several strengths. Both objective neuropsychological tests and subjective assessment were used to measure cognitive changes over time, a longitudinal design was employed with the last testing time point at one year after the start of chemotherapy, and the inclusion of a matched-control group. There are also some interpreted caveats that should be noted. Although selected carefully, some of the neuropsychological sub-tests used in the study may not have been sensitive enough to capture subtle cognitive changes induced by chemotherapy and this may have contributed to the inconsistencies between the neuropsychological test scores and self-reported cognitive complaints. On the other hand, the impact of such subtle cognitive changes on everyday functioning is likely minimal. A second caveat is that the 1-Year follow-up occurred one year after the start of chemotherapy; therefore, it may be possible that the findings might represent ongoing recovery at the 1-Year timepoint. The last caveat is that data were only collected in women with stage I-III breast cancer, so the conclusions cannot be extended to patients with other stages of breast cancer, with other types of cancer, or men.

In summary, chemotherapy-induced subjective complaints in neurocognitive functioning were reported by patients (compared to matched-controls) not only during chemotherapy, but even one year after treatment. On the other hand, there were no pre- to post-chemotherapy changes in objectively measured cognitive functioning, although there were improvements from both of those time-points to the 1-Year follow-up. The matched-controls, however, improved during all three intervals. One plausible explanation of this pattern is that chemotherapy may acutely diminish normative practice effects generally seen with repeated test exposures. Worsening objective cognitive function was predicted by increased disruption of CARs, increases in subjective complaints of neurocognitive functioning, decreases in sleep quality, and increases in time spent napping while worsening subjective reports of cognitive function were predicted by decreases in objective measures of neurocognitive functioning, and decreases in sleep quality, fatigue, and depression. Interventions targeting these predictors, such as light therapy for improving CARs,[51, 52] and fatigue [50], and cognitive behavioral therapy for insomnia for sleep [53], might benefit patients’ cognitive function during and after chemotherapy.
